# Droplet Manipulation under a Magnetic Field: A Review

**DOI:** 10.3390/bios12030156

**Published:** 2022-03-02

**Authors:** Gui-Ping Zhu, Qi-Yue Wang, Zhao-Kun Ma, Shi-Hua Wu, Yi-Pan Guo

**Affiliations:** Department of Aerospace Systems Engineering, School of Astronautics, Nanjing University of Aeronautics and Astronautics, Nanjing 210016, China; wqy2115@nuaa.edu.cn (Q.-Y.W.); zkk380@nuaa.edu.cn (Z.-K.M.); wushihuawushihua@outlook.com (S.-H.W.); guoyipan@nuaa.edu.cn (Y.-P.G.)

**Keywords:** microfluidics, magnetic field, magnetization, liquid actuation, droplet manipulation

## Abstract

The magnetic manipulation of droplets is one of the emerging magnetofluidic technologies that integrate multiple disciplines, such as electromagnetics, fluid mechanics and so on. The directly driven droplets are mainly composed of ferrofluid or liquid metal. This kind of magnetically induced droplet manipulation provides a remote, wireless and programmable approach beneficial for research and engineering applications, such as drug synthesis, biochemistry, sample preparation in life sciences, biomedicine, tissue engineering, etc. Based on the significant growth in the study of magneto droplet handling achieved over the past decades, further and more profound explorations in this field gained impetus, raising concentrations on the construction of a comprehensive working mechanism and the commercialization of this technology. Current challenges faced are not limited to the design and fabrication of the magnetic field, the material, the acquisition of precise and stable droplet performance, other constraints in processing speed and so on. The rotational devices or systems could give rise to additional issues on bulky appearance, high cost, low reliability, etc. Various magnetically introduced droplet behaviors, such as deformation, displacement, rotation, levitation, splitting and fusion, are mainly introduced in this work, involving the basic theory, functions and working principles.

## 1. Introduction

In recent years, the manipulation of droplets and cells [1,2,3] has attracted much attention in biomedicine, chemistry and hydromechanics, especially microdroplets. The volume of microdroplets is usually on the microliter scale, which is low in production cost and minor in consumption. With high yield and purity ensured by the closed environment, which is almost free from external pollution, microdroplets become a preferable chemical reaction container [4,5], which promotes their wide application in synthesis [6], detection, delivery and packaging of various reagents, drugs and particles [7,8,9], culture, transportation, isolation and dissolution of cells and seeds [10], separation of plasma [11], etc. Microdroplets are typically mass generated and manipulated in microfluidic devices. The development of microelectromechanical systems (MEMS) technology enables the production of more powerful microfluidic devices, making the operation of microfluidic processes flexible. Meanwhile, with the development of computational fluid dynamics (CFD), the study of microdroplets is facilitated with numerical methods that contribute to robustness in high efficiency, low cost, underlying mechanism and so on.

Until now, microfluidic technology has been developed for a variety of droplet control methods coupled with light, sound, heat, electric field, magnetic field, pneumatics and mechanical devices to transport, sort, split, merge and deform droplets [12]. Among all the developed microfluidic strategies, the magnetic method is a prospering research area, which is very popular due to its remarkable advantages in remote and instant control of droplets. In addition, precise and localized manipulation of droplets is ensured by the programmable magnetic field distribution and thus the exerted force by adjusting the current of the electromagnetic coil or the position of the permanent magnet. Overall, the performance of the magnetic droplet manipulation is influenced by numerous factors, including liquid properties, surrounding medium, the interfacial characteristics between the immiscible fluids, the wetting behavior, the structure of the device, the configuration and tuning of the magnetic field and so on.

Various operations of droplets have been performed under a magnetic field, such as ferrofluid droplet stretching [13,14,15,16,17], deflecting [18,19], sorting [20,21], merging [14,22,23,24,25] and splitting [26,27,28,29], as well as the application in mixing chemical reagents [30], capsule synthesis [31], microlens [32], etc. Based on droplet manipulation, functional group-modified magnetic nanoparticles (MNPs) [33] dispersed in the carrier fluid are used for oil contamination separation [34], oil and gas processing [35], chemical extraction [36] and detection and extraction of heavy metal ions [37] under the magnetic field. With the development of magnetic manipulation of droplets and nanoparticles in molecular biology, MNPs further fulfilled the manipulation of the attached biological macromolecules, such as DNA [38,39], RNA [40,41], antibodies, proteins [42], enzymes [43,44] and even viruses [45]. Ferrofluid and the constituted MNPs are also widely used in drug delivery [46] and release [47], immunoassay [48], targeted therapy [49], biosensor [50], magnetic actuator [51,52,53], mechanical seal [54] and so on.

Ferrofluid is the most commonly used magnetic fluid, which is a colloidal dispersion synthesized by the suspension of MNPs (normally around 10 nanometers in diameter) in a carrier liquid [55], which exhibits the fluidity of liquid materials and the magnetic properties of solid materials. Thanks to paramagnetism, MNPs can be magnetically manipulated because of their large surface area and volume ratio. The precipitation and aggregation of MNPs are effectively prohibited by the thermal Brownian motion and surfactant ingredient in ferrofluid. Upon applying a magnetic field, ferrofluid is affected by the magnetic force exerted on individual MNPs and volumetric magnetic force determined by the field flux density, gradient, liquid susceptibility, droplet volume and so on. The approximated volumetric magnetic force is proposed for the relatively small volume of the manipulated droplets [56]. The magnetization is governed by the Langevin function in terms of magnetic susceptibility, which varies with the magnetic field [57]. Furthermore, the magnetic force on each particle is derived and presented for MNPs with shapes in regular spheres [58,59]. In addition, energy analysis in the magnetic material reveals the heat exchange or electromagnetic radiation in terms of the magnetic field characteristics, the magnetic susceptibility and the liquid volume [60]. With the magnetic material immersed in an instantly enhanced or weakened magnetic field, there will be generation or loss of heat applicable for cryogenic techniques exploring or temperature adjusting in ferrofluid. In numerical calculation, the interface of multiphase flow can be tracked by means of volume fraction method (VOF) [61], level set method (LSM) [62], phase field method (PFM), dynamic mesh method (DMM), etc. In microgravity, the force balance of the ferrofluid droplet is identified by magnetic force, resistance [63] and interfacial tension [64].

To sum up, magnetic tuning of liquid droplets in microscale provides a reliable approach for sample handling in numerous scientific pursuits. This work aims to provide a selected review of the progress of droplet manipulation in an external magnetic field. The accomplishment in this field is mainly summarized and categorized based on different basic processes discussed in the following sections, together with typical achievements and examples of their applications.

## 2. Droplet Manipulation in a Magnetic Field

There are many ways to control microdroplets, including optical [65], thermal [66], acoustic [67], electric [68], magnetic methods [69], etc. Among them, the magnetic approach has the advantages of remote control without sensitivity to pH and dielectric properties of the liquid medium. The high-throughput handling of droplets was achieved together with the extraction of magnetic materials due to the adsorption and magnetic actuation function of MNPs in ferrofluid [70,71]. The magnetic force on MNPs or ferrofluid droplets varies with the magnitude and direction of magnetic intensity. The regulation of magnetic control facilitates droplet processes, including generation, deformation, motion, transport, fusion and splitting, sorting and so on. Electromagnet needles [72] and permanent magnets [73] are both capable of manipulating magnetic droplet transport, fusion and dispersing. Moreover, Park et al. [74] flexibly used a permanent magnet to transport and merge magnetic droplets in a 3-dimensional space. With the development of MEMS technology, complex electronic components were integrated on microfluidic chips for droplet programming [75]. The magnetically induced manipulation is thus presented in this work according to the droplet process with analysis on liquid properties, channels and field configurations, etc.

### 2.1. Droplet Generation

Droplet generation is a prerequisite for microfluidic manipulation. Preferred due to high reliability and low cost, passive methods are widely explored for operating purely based on the channel configurations and liquid properties, while active methods are developed taking advantage of an external force field to overcome the issues of low efficiency and throughput. The active approach is implemented with microfluidic devices commonly coupled with magnetic fields, lasers, electric fields and ultrasonic waves to realize and regulate the generation of droplets [76] directly applicable in inkjet printing, metal 3D printing, capsule synthesis, etc.

In magnetofluidic droplet generation, a magnetic field raised by electromagnetic coils or permanent magnets is applied mainly to control the droplet generation rate, size and shape. The generation process, as well as the resulting droplet frequency, size and interval, are governed by the continuity equation and the Navier–Stokes equation with the volumetric force, including inertial, viscous, interfacial, gravitational and externally applied magnetic force [77]. For a physical understanding, the significance of the forces is usually analyzed by the nondimensionalized number [78]. In Table 1, the most commonly adopted dimensionless number is illustrated by Reynolds, Capillary, Weber, Bond and Magnetic Bond numbers, specifically for magnetic droplet generation. From the definition of the number, Re, Ca, We and Bo are calculated in terms of average fluid velocity (u), density (ρ), characteristic dimension (R, radius or contact radius), dynamic viscosity of the liquid (μ), gravity acceleration (g), interfacial tension (σ) and the volume mass difference between the continuous and dispersed phase (Δρ). For the magnetic approach, Bm is determined by the permeability of the vacuum ($\mu_{0}=4\pi \times 10^{-7}NA^{-2}$), susceptibility of the magnetic fluid (χ), volume of the droplet (V) and the applied magnetic field intensity ($H_{0}$).

With different flow rate ratios, the droplet generation process is typically defined as slug flow, dripping flow and jetting flow with or without a magnetic field. The flow pattern and ferrofluid droplet generation can be illustrated in terms of the capillary number of the continuous phase and the Weber number of ferrofluids [78]. The low capillary number makes a slug flow when the continuous phase flow rate is relatively small. In this case, ferrofluid completely occupies the channel and leads to an increment in upstream pressure. As the flow rate of the continuous phase increases, a dripping flow pattern is formed with viscous force playing a prominent role in droplet breakup. With a continuous phase flow rate much larger than that of ferrofluid, the effect of capillary instability gives rise to the jetting flow regime. With an increment in magnetic strength, the flow tends to be a dripping pattern. The induced magnetic force acts on the head of the ferrofluid and thus decreases the length of the generated droplet. However, the magnetic field plays a negligible role in the transition between dripping and jetting flow [79].

**Figure 1:**
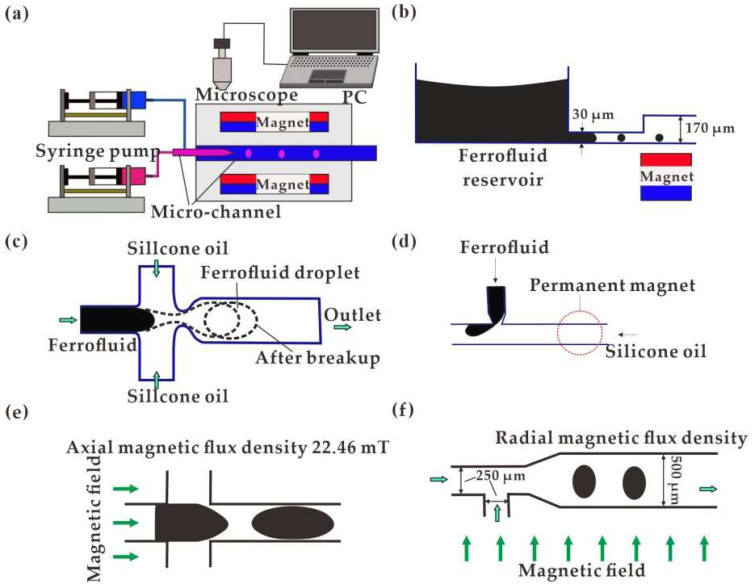
Ferrofluid droplet generation devices: (**a**) Straight channels with permanent magnetic field [80]; (**b**) Driven by a permanent magnet without a pump [81]; (**c**) In cross-shaped channel with a perpendicular uniform magnetic field [88]; (**d**) In T-shaped microfluidic chip with a permanent magnet [84]; (**e**) Horizontal magnetic field [90]; (**f**) Radial magnetic field [91].

Conventional straight channels or nozzles were commonly employed for droplet generation directly as shown in Figure 1a. By coupling with a permanent magnet, the tunability of the ferrofluid droplet generation was realized by the non-uniform magnetic field with strength high in the center and weak at both sides [80]. The generation rate of ferrofluid droplets was increased as the droplet stretched forward. Without external syringe pumping, Kahkeshani et al. [81] proposed the generation of ferrofluid droplets in a straight-channel under the driving of the gradient magnetic field, as shown in Figure 1b. In addition, a perpendicular uniform magnetic field was applied for the numerical study of ferrofluid droplet generation in a straight-channel microfluidic device [82]. The flow velocity increment and droplet size declination were investigated on a larger magnetic bond number.

Other typical microfluidic devices were proposed to control ferrofluid droplet generation in T-shaped [83,84,85] and cross-shaped [10,86,87] channel structures coupled with an electromagnetic field. For cross-shaped chips with a magnetic field applied along the flow field, the diameter of the ferrofluid droplets would become larger as the magnetic intensity gradually increases at a constant flow rate. On the contrary, higher magnetic intensity results in a smaller droplet size for the T-shaped chip. The process of droplet generation in the cross-shaped and T-shaped microfluidic chip is respectively shown in Figure 1c [88] and Figure 1d [84]. Alternatively, a permanent magnet was positioned above the T-shaped channel for generation of ferrofluid droplets with the reduction in generation frequency and increment in curvature radius attributed by the permanent magnetic field [85].

Except for the magnetic intensity, the shape and size of the generated droplets are related to the flow rate of the liquid sample, as well as the direction of the applied magnetic field. Generally, a larger flow rate and stronger magnetic field would accelerate the breaking up of the ferrofluid droplets, giving rise to a smaller volume. For a straight vertical nozzle structure, Fabian et al. [89] investigated the dripping of ferrofluid under a horizontal or vertical magnetic field, which contributed to larger or smaller ferrofluid droplets, respectively. Wu et al. [90] systematically studied ferrofluid droplet generation in a cross-shaped channel under a transverse (perpendicular to liquid flow) or a longitudinal (parallel to the liquid flow) magnetic field. In the transverse case, the generation rate was increased with the length of ferrofluid droplets shortened. In the longitudinal case, droplet generation slowed down with the length elongated, as shown in Figure 1e. For a T-shaped channel under a transverse magnetic field, the interval of the generated ferrofluid droplets is larger at a stronger field strength [91], Figure 1f.

Furthermore, programmability was implemented for non-magnetic water droplet generation in ferrofluids with rotating permanent magnets [92] and metal droplet generation under the action of Lorentz force formed by an electromagnetic field [93]. In brief, the size and shape of generated droplets, as well as the generation rate, are related to characteristic parameters, such as the flow rate, surface tension, magnetic permeability, the applied magnetic field, etc.

### 2.2. Droplet Deformation

**Figure 2:**
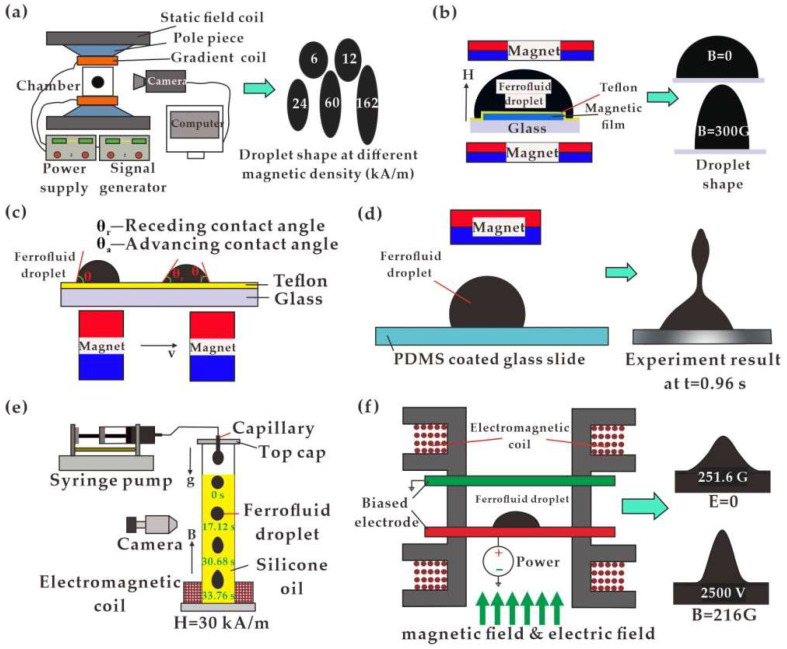
Suspended and sessile ferrofluid droplet deformation in a magnetic field: (**a**) Suspended ferrofluid droplet undergoes tensile deformation [15]; (**b**) Sessile ferrofluid droplet with magnetic dots [95]; (**c**) Sessile ferrofluid droplet above permanent magnet [101]; (**d**) Sessile ferrofluid droplet beneath permanent magnet [102]; (**e**) Asymmetrical deformation with gravitational force [105]; (**f**) Ferrofluid peak with coupling of magnetic and electric fields [107].

Once immersed in a magnetic field, magnetic forces on MNPs or magnetic liquid cause various deformations of the magnetofluidic droplets. The suspended ferrofluid droplet undergoes tensile deformation along the direction of the applied magnetic field [13,14,15,16,17]. Normally, the droplet appears to be stretched into a larger aspect ratio in a stronger magnetic field (Figure 2a) [15]. For sessile droplets, the vertically applied magnetic field forces the droplet to undergo deformation or even instability [94]. As shown in Figure 2b, Lee et al. [95] applied magnetic dots with a diameter of 0.5–0.95 times that of the sessile ferrofluid droplet to analyze the aspect ratio of the droplet in terms of magnetic field strength. In addition to profile analysis, wettability of the droplet [96] has been extensively studied in terms of contact angle variation [97,98,99,100]. A permanent magnet was placed under the sessile droplet, where the contact angle gradually decreased as the magnetic intensity increased [101]. Upon reaching a critical condition, the sessile droplet incepted and slid continuously with the moving permanent magnet, with the dynamic advancing and receding contact angles shown in Figure 2c. For the permanent magnet placed above, the droplet on a hydrophobic substrate was stretched and even split along the vertical direction with contact angle decreasing at increasing field strength, as shown in Figure 2d [102]. The shape deformation and the splitting process were determined by the combined effects of surface tension and magnetic force, which were also affected by nanoparticle concentration.

The direction of the droplet deformation is consistent with the magnetic force, which directs to a higher value of magnetic flux density. Ghaderid et al. numerically studied the influence of the vertical magnetic field on the droplet falling process [103]. The magnetic forces symmetrically distributed on the upper and lower interface, which can intuitively explain the suspended droplets’ uniform tensile deformation to both ends. Haberad et al. [104] numerically calculated the magnetic field distribution around the droplet immersed in a uniform magnetic field and further verified the working mechanism. Due to magnetization, a strong gradient of magnetic intensity was generated at both ends of the droplet without significant fluctuations in the center area. For ferrofluid droplets in a magnetic field with non-negligible gravitational force, the droplets would have asymmetrical deformation during the falling process [105]. The shape of the droplet went through the initial oblate ellipsoid to a sphere, then to a long ellipsoid and finally to a teardrop shape, as shown in Figure 2e. In addition to the above-mentioned meniscus under a magnetic field only, Jackson et al. [106] proved the ability to acquire ferrofluid peaks with coupling of magnetic and electric fields, as shown in Figure 2f. Furthermore, sessile ferrofluid droplet deformation was studied under the control of the magnetic field in conjunction with gravity and sound [107].

On the basis of magnetic droplet deformation and contact angle shifting in a magnetic field, various applications have been fulfilled, such as microfluidic actuators [52,108] and drive other transparent liquid via communicating vessels for making adaptive liquid microlens, grating, etc. [109]. With an electromagnetic field, the deformation of magnetic sessile droplets would lead to an adjustable droplet microlens by connecting droplets through a channel [110] or cavity [111]. The liquid properties of ferrofluid and the characterization of the external field play essential roles in the deformation of the magnetic droplet, and thus the performance of the lens. For miniaturization, a microcoil array [112] was adopted to actuate ferrofluid droplets for precise fabrication and easy integration.

### 2.3. Droplet Transportation

Magnetically induced droplet transportation enables reliable sample handling for bioassays, drug preparation, chemical reactions and so on. The magnetically actuated droplet motion can be regulated as predefined specific trajectories, such as rectilinear motion and rotational motion. The magnetic field configuration plays a vital role in the actuating and driving of magnetic droplets, including distribution, field strength and field gradient. The motion of ferrofluid droplets in a gradient magnetic field has been extensively investigated with the magnetic component performing as valve and actuator to pump other immiscible liquids. Normally, the pumping performance was tuned and optimized with a wise determination of channel configuration and magnetic properties. In a trapezoidal silicon microchannel, the driving of ferrofluid droplets was applied for liquid sampling in a magnetic field gradient generated by a permanent magnet with a stepping motor [113] and an array of electromagnets [114].

**Figure 3:**
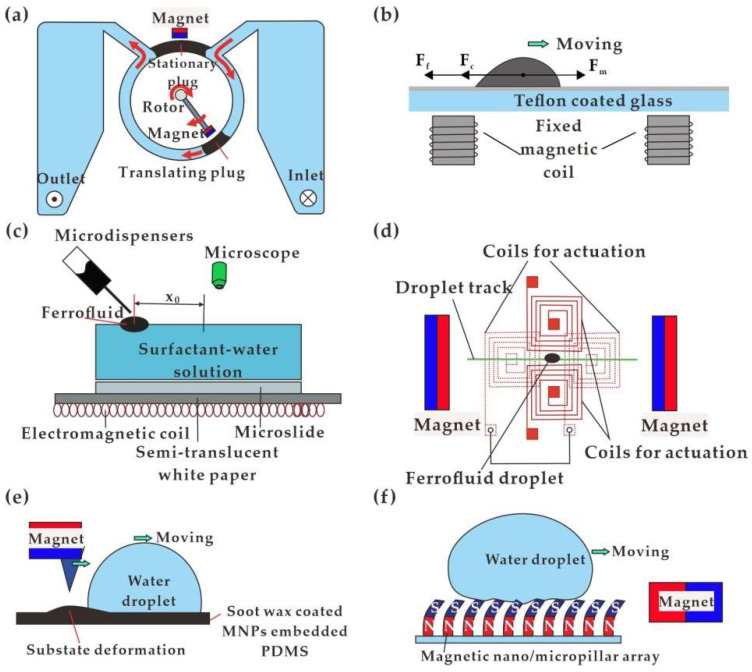
Droplet transportation in magnetic actuation: (**a**) Ferrofluid plugs with rotating permanent magnets [115]; (**b**) Ferrofluid droplet with sliding permanent magnets [73]; (**c**) Ferrofluid droplets with electromagnetic coils [56]; (**d**) Ferrofluid droplets with permanent and electromagnetic fields [122]; (**e**) Water droplet moving on substrate deformation [128]; (**f**) Water droplet moving based on magnetic nano/ micropillar arrays [130].

By applying only permanent magnets, Hatch et al. [115] proposed a droplet handling strategy for the pumping of immiscible continuous phases in a ring channel by manipulating ferrofluid plugs with rotating permanent magnets (Figure 3a). For a systematic understanding of the pumping behavior, the flow rate was studied with various chip dimensions and the speed of magnets directing the ferrofluid droplets. With minimal backpressure, the maximum flow rate and pressure head obtained were 45.8 μL/min and 1.2 kPa, respectively. In the originally adopted circular channel configuration, ferrofluid served as a rotating or sealing component in the pump with plunger and blade shape. With a similar design of rotary magnet driven by a motor, Fu et al. [116] proposed pumping of diamagnetic fluids by ferrofluid working as the plunger of the pump. The rotational motion of the ferrofluid droplets was realized [117] and further demonstrated for droplet-based PCR [118,119]. In conventional straight microchannels, multiple [120] ferrofluid droplets were introduced and tested for pumping of the DI water. In digital microfluidics, the permanent magnet was robust in droplet wetting properties shift, deformation, continuous sliding, and thus the fusion and split processes (Figure 3b) [73].

To avoid the pinching moment of the droplet under moving magnets, a magnetic coil was adopted in investigating various volumes of ferrofluid droplet (5 μL to 150 μL) transportation with a bio-compatible surfactant [121]. The stationary coil was beneficial in preventing the disengagement of the droplet during the sliding process, which possibly induced difficulties in predicting the exact trace line [101]. The platform was examined for its versatility in droplet transport both on a hydrophobic solid substrate and immersed in olive oil, with various magnetic flux densities, droplet volume, duty cycles and frequencies. For further precise position and manipulation of droplets at a distance from the magnets, an array of micro-coils were enormously developed and optimized for single or multiple droplet transportation in the demanded trajectory. Chakrabarty et al. [56] applied a gradient magnetic field generated by an embedded micro-electromagnetic coil to drive 0.5 μL ferrofluid droplets on the surfactant–water solution moving toward the center in a linear or predefined meandering path. A single planar squire coil formed a magnetic field with strength owning the maximum value at the center and dissipating sharply toward the periphery. The sequentially switched array of square spiral microelectromagnets facilitated the sophisticated regulation of droplet sliding paths (Figure 3c).

The superposition with a permanent magnetic field offers versatility in droplet actuation in an electromagnetic field. On a digital microfluidic platform, Beyzavi et al. [122] designed a magnetic field generated by two pairs of electromagnetic coils to restrain the ferrofluid droplets (0.5~3 mm in diameter) from moving in the horizontal direction, as shown in Figure 3d. The superposition with a pair of permanent magnetic fields was necessary for creating a single field maximum for droplet attraction. The additional permanent magnetic field also helped in stronger field strength, which induced a larger peak velocity of the droplet [123]. Probst et al. [124] sequentially powered four electromagnetic coils to realize the spiral motion of ferrofluid droplets. The magnetic remote control provided an approach for precise positioning, as well as steering along any desired 2-dimensional path of a single ferro fluid droplet. In addition to ferrofluid, droplet transportation was realized for magnetic liquid marbles by a permanent magnet [125] and EGaIn droplets by the generated Lorentz force [126].

An alternative option of magnetic actuation is a functioning substrate for working as a driving component. Inspired by deformable paramagnetic liquid substrate [127], Damodara et al. [128] utilized a permanent magnet to achieve droplet movement on the MNP-embedded PDMS chip, as shown in Figure 3e. Similarly, a superhydrophobic magnetic elastomer [129] was developed with surface depression under the action of a magnetic field to accomplish the motion of a non-magnetic droplet. Magnetic nano/micropillar arrays (MNA) [130] were proposed to transport droplets through pillar bending in a permanent magnetic field, as shown in Figure 3f. By coupling with gravitational force, Wang et al. [131] concluded that the reduction in height and shift in motion trace of the droplet rebound in a magnetic field.

### 2.4. Droplet Sorting

Droplets generated in all kinds of devices need to be sorted for an experiment or industrial production with specific requirements, such as speed, size, material and composition. According to the driving mechanism, droplet sorting is categorized into passive and active droplet ways [132]. The passive method sorts droplets by taking advantage of the physical or chemical properties of droplets, such as speed, interfacial tension [133], size [134], viscosity [135], etc. However, this sorting method has a shortage in delay and slow response. The active method mainly sorts droplets by utilizing an externally applied driving source, such as pneumatic actuator, ultrasonic, thermal, electric and magnetic force [3].

**Figure 4:**
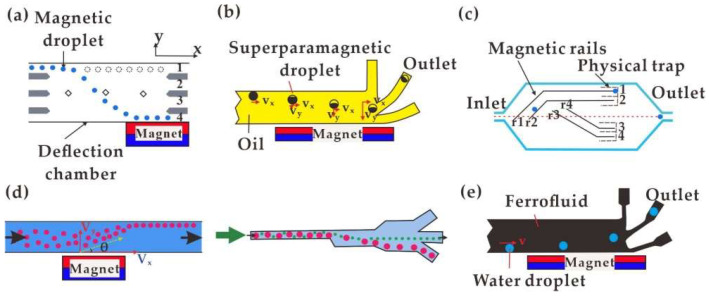
Different magnetic sorting methods: (**a**) Based on the difference in magnetism [136]; (**b**) Droplets deflected to the indicated outlet [18]; (**c**) Magnetic rails [21]; (**d**) Based on the difference in flow speed and droplet size [19]; (**e**) Deflect of water droplets in ferrofluid medium [137].

Magnetic active control is an instant and efficient way to sort ferrofluid droplets or magnetic particles. According to the difference in magnetism, Figure 4a shows a ferrofluid droplet sorting method using a permanent magnetic field [136]. During the sorting process, the droplet would be deflected to different extents, varying with the size of magnets placed under the microfluidic channel and the magnetic particle concentration in ferrofluid. A similar device was adopted to transport single or batch superparamagnetic droplets with their speed reaching 10 per second (Figure 4b) [18]. To sort droplets with magnetic beads, Teste et al. [21] developed a magnetic rail for steering the droplets along the designed track (Figure 4c). In addition, ferrofluid droplets were deflected to different extents in terms of flow speed or droplet size (Figure 4d) [19]. Hydrophobic ferrofluid was applicable in wrapping water droplets for indirectly sorting non-magnetic water droplets in a magnetic field [137]. Water droplets were deflected to flow out along three different passages by continuously moving the horizontally positioned permanent magnet (Figure 4e).

Magnetically induced droplet sorting is mainly applied in life science, bioengineering, medicine, diagnosis and medical testing, etc. By successfully encapsulating biological reagents or chemical samples in droplets, sorting of cells in microfluidic chips offered a promising way for disease detection and analysis [138]. With technology developed for labeling with superparamagnetic iron oxide nanoparticles, cell detection, loading, delivering and further sorting are ensured in all kinds of biomedical tasks [139]. In addition, Sung et al. [140] magnetically sorted droplets containing different microalgae cells with various densities to extract microalgae with high growth performance. Compared with conventional methods, magnetic droplet sorting is characterized by high throughput and purity, zero contact pollution, low sample consumption and cost, but a relatively slower response. Another fundamental drawback is the need for magnetic components for interaction with the externally applied magnetic field [141,142]. Moreover, the labeling of non-magnetic target droplets by magnetic material such as MNPs may give rise to the variation of physical and chemical properties, which could undermine the chemical/biological compatibility.

### 2.5. Droplet Coalescence and Splitting

**Figure 5:**
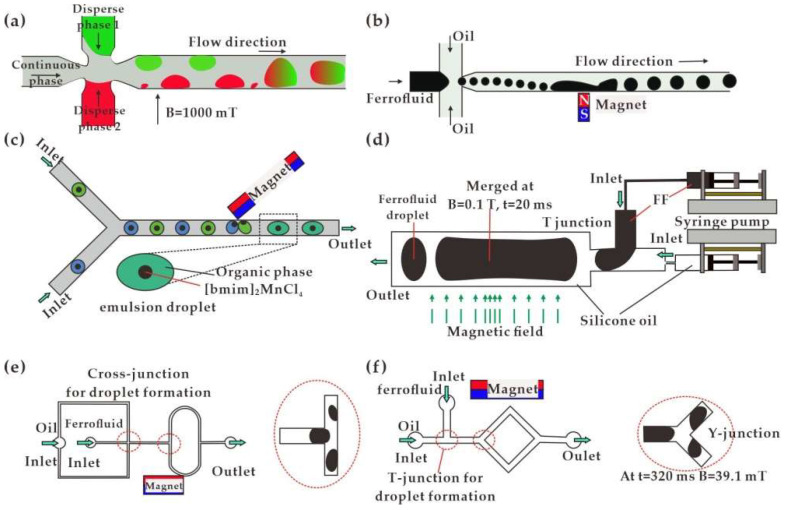
Droplet coalescence and splitting in a magnetic field: (**a**) Uniform magnetic field induced droplet merging in a cross-shaped channel [22]; (**b**) Permanent magnet induced droplet merging in a cross-shaped channel [23]; (**c**) Permanent magnet induced droplet merging in a Y-shaped channel [24]; (**d**) Droplet merging in coupling uniform and non-uniform magnetic fields [25]; (**e**) Droplet splitting in T-shaped channel [26]; (**f**) Droplet splitting in Y-shaped channel [27].

The process of droplet coalescence or splitting in the microfluidic channel can be actively controlled by a magnetic field. In the microfluidic platform, magnetically induced droplet coalescence can be classified into the following two categories according to the working principle. Through controlling the flow moving direction and flow rate, Varma et al. [22] applied a 1000-mT uniform magnetic field to droplets in a cross-shaped channel. The speed of the droplets changed abruptly, resulting in the coalescence of two different types of droplets (Figure 5a). A permanent magnet was also functional for flowing droplets deflecting toward the magnet and merging into a larger droplet in the cross-shaped channel (Figure 5b) [23]. The non-uniform magnetic field was formed with a peak value of 50 mT. Similarly, a permanent magnet was placed in direct contact with the top surface of a Y-shaped channel to magnetically realize the assembling of double emulsion droplets near the magnet and then coalescence, as shown in Figure 5c [24].

Alternatively, droplet coalescence would be accomplished by profile deformation in an external magnetic field. In a uniform magnetic field, Ghaffari et al. [14] studied the coalescence of two falling ferrofluid droplets based on the fundamental understanding of pendant droplet deformation in terms of field intensity, magnetic susceptibility, surface tension and droplet size (Figure 5d). The analysis was presented with droplet aspect ratio as a function of magnetic bond number and susceptibility. The numerical results, together with the verification by comparison with experimental data, indicated a magnetic approach to be beneficial in both coalescence and breaking of emulsion. In a T-shaped microchannel, the combination of uniform and non-uniform magnetic fields was utilized to explore multiple ferrofluid droplet actuation, inter-droplet space tuning, droplet deformation and merging [25].

In addition, the microfluidic droplets would be split when passing through the T-shaped [26,143,144,145], λ-shaped [146] and Y-shaped [27,147] channels. As shown in Figure 5e,f, when a permanent magnet is placed on one side of a T-shaped [26] or Y-shaped [27] channel, there would be an asymmetric splitting of the droplet with size and frequency programmable through manipulating the field. A stronger non-uniformly distributed magnetic field facilitates the non-breakup of droplets at a relatively small flow rate ratio between the continuous and dispersed phase. Chen et al. [28] proposed splitting of the magnetic droplet by adopting an orifice under the attraction of an electromagnetic field. The droplet characteristics, such as the sizes of the daughter droplets and the stretching lengths, were interpreted according to orifice diameter and local field strength. By droplet merging, quantitative methylation-specific PCR was realized in a microfluidic device with three identical and parallel lanes [148]. Droplets containing human cell suspension underwent bisulfite conversion and cell lysis through droplet coalescence. In addition, further merging with secondary droplets led to DNA binding to magnetic particles for further processing.

Besides the above-mentioned continuous microfluidic process, droplet coalescence and merging are also widely explored and analyzed in digital microfluidic devices with droplets deposited on an open platform. By inducing magnetic hydrodynamic instability, a large mother ferrofluid droplet on the magnet disc arrays was uniformly split into several small daughter droplets with the lattice structure affected by the field configuration [29]. Koh et al. [149] utilized a permanent magnet to drive the ferrofluid droplet fusion with the water droplet on the Teflon-coated substrate. The control of oil-based ferrofluid droplet transportation and fusion with a diamagnetic oil droplet was realized on superhydrophobic ZnO nanorod arrays by adopting an external magnetic field [150].

During the coalescence and splitting process, droplet sizes and flow rate can be precisely adjusted by the magnetic field to satisfy the requirement of further investigation. Fusion and splitting of microdroplets have broad applications in chemical reagent synthesis and dispersion, capsule synthesis and pharmaceutical detection, etc. By droplet splitting, Lehmann et al. [39] implemented the purification process by using a coil matrix to manipulate MNPs for extracting DNA from cell samples. In a permanent magnetic field with a peak at 400 mT, a magnetic Janus droplet was synthesized in the cross-shaped channel [151] and further applied for protein detection [152,153]. Furthermore, Alorabi et al. [31] successfully synthesized multi-layered capsules for drug delivery by controlling the magnetic droplets flowing into the polyelectrolytes under a magnetic field.

### 2.6. Droplet Levitation

**Figure 6:**
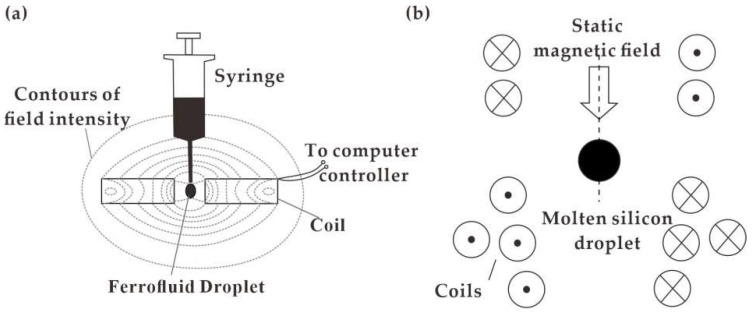
Magnetic levitation of droplets: (**a**) Ferrofluid droplet levitation and splitting in an electromagnetic field [154]; (**b**) Molten silicon droplets levitation in a coupled field [161].

Droplet levitation achievable under a magnetic field exhibits relatively stable equilibrium without media contact. Undoubtedly, the static magnetic field can levitate paramagnetic medium based on precise regulation. Both experimental and numerical results were presented for ferrofluid droplet levitation by an electromagnetic coil. The droplet dynamics were studied as a function of the applied magnetic field, including equilibrium shape and oscillation, as shown in Figure 6a [154]. It is worth noting that magnetized diamagnetic liquid and its levitation are of great importance in industrial and scientific fields. In terms of the propulsion working principle, the magnetic levitation of diamagnetic droplets was introduced with static and alternating magnetic fields individually.

The levitation of diamagnetic droplets offers the capability of investigating thermal characteristics, surface properties and hydrodynamics, sophisticated convection and so on. For levitation of diamagnetic liquids (such as water and alcohol), the field is required to have characteristic parameters large enough in both strength and gradient magnitude. Commonly, a strong static magnetic field generated by a superconducting coil was utilized to realize the suspension of diamagnetic liquid. In the preliminary work, Beaugnon et al. [155] used a super-static magnetic field of up to 20–27 T to levitate weakly diamagnetic liquids such as alcohol, water, acetone, etc. By being immersed in a magnetic field gradient, liquids were forced to lower field regions under the effect of a driving force stronger than gravity. The technique is applicable in biological fields, as most organic materials have a diamagnetic susceptibility almost in the same order of magnitude as water [156]. In addition, Liu et al. [157] used a 17 T static magnetic field to levitate a large water droplet with a few mm in diameter. The generated magnetic force follows $B\left(z\right)\nabla B\left(z\right)=-\mu_{0}\rho g/\left|\chi \right|$, where B(z), $\mu_{0}$, χ and **B** are the vacuum permeability, the magnetic flux density in the vertical direction, the magnetic susceptibility of the droplet and the externally applied magnetic flux density [158,159]. Therefore, the droplet is levitated by the generated repulsive magnetic force balancing the volumetric gravitational force. To fulfill the working mechanism on magnetism, Pang et al. [160] designed experiments for observing the levitation and interaction of pure water droplets in a superconducting magnetic field with strength up to 16.1 T. The suspension state significantly relied on the interaction between the superconductive magnetic field and magnetized water droplets.

In addition, an alternating magnetic field is adopted to levitate conductive liquids such as molten metal. Commonly, an AC coil is applied to generate the magnetic field and thus results in the Lorentz force as the driving source. It is worth mentioning that there will be generation of eddy current and heat in the droplet. The coupling of an AC coil with a static magnetic field was capable of levitating molten silicon droplets, as shown in Figure 6b [161]. The oscillation, equilibrium, deformation and temperature field of the droplet were systematically interpreted in terms of the static field strength ranging from 1-5 T and the Marangoni effect. The same group numerically studied the flow and temperature field distribution of molten copper droplets levitated by an electromagnetic approach [162]. The influence of the eddy effect was considered in the droplet and coils for taking into account the non-uniformity of current density. The eddy effect gave rise to higher magnetic force, velocity and unevenly distributed temperature in the levitation process. In microgravity, Bojarevics et al. [163] performed a numerical simulation on the melting of silicon droplets in an AC+DC magnetic field and presented the typical temperature and velocity field. The stable droplet suspension helped in studying heat transfer in microgravity environments [164], ensuring demanding chemical reactions without bacterial and virus pollution, smelting high-purity materials [165], promoting the fusion of incompatible alloys [166] and optimizing the welding effect [167].

## 3. Conclusions and Future Directions

Through the above analysis, we witnessed the development of magnetically induced droplet manipulation and its application prospects. As one of the most critical issues in the microfluidic area, magnetic control exhibits the most vital ability to wirelessly manipulate droplets constituted with biological reagents or chemical samples. In terms of fabrication and integration, magnetic manipulation is relatively easy to implement with advantages in locality, non-contact, strong anti-interference, etc. A variety of scientific works are presented in this review. The recent achievements and advances are categorized and discussed in terms of different processes, including droplet generation, deformation, transportation, sorting, fusion, splitting and levitation. Other specific functions are given by extracting magnetic particles in droplets, sorting droplets with magnetic differences, driving or transporting droplets, smelting metal, etc. Microfluidic devices coupled with wisely designed and regulated magnetic fields have unique advantages in biomedical and chemical applications, which can manipulate cells with natural magnetism, label non-magnetic target cells with MNPs, encapsulate cells or viruses, culture cells, sort cells, synthesize drug capsules, detect cancer and conduct targeted therapy. Overall, the magnetic control of droplets can be achieved by regulating fluidic properties, channel and magnetic field configuration to precisely tune the liquid wettability, droplet profile and size, droplet intervals, etc. The development of magnetic droplet manipulation can promote cross-disciplinary cooperation and exchange, which break the barrier between disciplines and provide novel vision in scientific research.

On the other hand, it is also worth mentioning that the defects of the magnetic approach are closely related to field configuration and material properties and need further excavation and improvement. The main characterization of the general performance in droplet handling includes response time and throughput, ease of fabrication, sample and device cost, system lifetime, accuracy and so on. Some of the interesting research directions and prospects are stated in the following section. Magnetic actuation is preferred in certain applications due to its advantages, such as easy fabrication and low cost. But to what extent magnetic droplet manipulation could contribute to the industry or biotechnology revolution would still depend on the response to the challenges. The integration of all components on the same platform demands the miniaturization of functional devices, such as pumps, valves, mixers, etc. The compromise would be necessary for the introduction of efficient active methods unfavorable in miniaturization and reliability, or passive methods with easy integration but less efficiency. We envision that the growing complexity of microfabrication, synthesis and assembly of composite materials would be required to support the adequate functions of the magnetic platform. For magnetic handling in biotechnology, the volume reduction of the macroscopic magnetic environment to microscopic cases in certain applications would be another considerable challenge, especially for droplet levitation with superconducting magnets. The molecules and MNPs in miniaturized systems also need to deal with the issue of adherence to boundaries due to the large surface/volume ratio or difficulty in entering microchannels due to the effect of capillary forces.

In addition, the experimental investigation requires further efforts on massive handling, instant tracking and monitoring. The manipulation of droplets would depend on technology for addressing information to identify and code a single droplet or a series of droplets. The addressing technology is foreseen to be a remote way realized by integrating components in the handling platform. The compatibility of magnetic handling with digital processing facilitates the programmability and diversity of droplet identification and treatment. Furthermore, a multi-functional droplet platform needs to be explored to provide complex, generic and robust handling. The application would offer integrated chemical, biological and medical functionality with specific strategies adopted. To conclude, on the basis of current accomplishments on the topic of droplet manipulation under magnetic field, this work summarized valuable theories and experimental behaviors, together with potential application prospects and challenges, in the hope of contributing to the construction of a sound and rigorous research structure that would be beneficial both for the understanding of its comprehensive working mechanism and for the future application and commercialization of this technology.

## Figures and Tables

**Table 1:** Dimensionless number in magnetic droplet generation.

Dimensionless Number	Formula and Physical Description
Reynolds number	Re=ρuRμ=Inertial forceViscous force
Capillary number	$Ca=\frac{\mu u}{\sigma }=\frac{Viscous\;force}{Interfacial\;tension}$
Weber number	$We=\frac{\rho u^{2}R}{\sigma }=\frac{Inertial\;force}{Interfacial\;tension}$
Bond number	$Bo=\frac{\Delta \rho gR^{2}}{\sigma }=\frac{Gravitional\;force}{Interfacial\;tension}$
Magnet Bond number	$Bm=\frac{\mu_{0}\chi V^{1/3}H_{0}^{2}}{2\sigma }=\frac{magnetic\;force}{Interfacial\;tension}$

## Data Availability

Not applicable.
